# A Maternal High-Fat Diet during Early Development Provokes Molecular Changes Related to Autism Spectrum Disorder in the Rat Offspring Brain

**DOI:** 10.3390/nu13093212

**Published:** 2021-09-16

**Authors:** Kinga Gawlińska, Dawid Gawliński, Małgorzata Borczyk, Michał Korostyński, Edmund Przegaliński, Małgorzata Filip

**Affiliations:** 1 Maj Institute of Pharmacology Polish Academy of Sciences, Department of Drug Addiction Pharmacology, Smętna Street 12, 31-343 Kraków, Poland; kingaw@if-pan.krakow.pl (K.G.); przegal@if-pan.krakow.pl (E.P.); mal.fil@if-pan.krakow.pl (M.F.); 2 Maj Institute of Pharmacology Polish Academy of Sciences, Laboratory of Pharmacogenomics, Department of Molecular Neuropharmacology, Smętna Street 12, 31-343 Kraków, Poland; gosborcz@if-pan.krakow.pl (M.B.); michkor@if-pan.krakow.pl (M.K.)

**Keywords:** autism spectrum disorder, ASD, DNA methylation, frontal cortex, high-fat diet, HFD, hippocampus, maternal diet, offspring, RNA-seq

## Abstract

Autism spectrum disorder (ASD) is a disruptive neurodevelopmental disorder manifested by abnormal social interactions, communication, emotional circuits, and repetitive behaviors and is more often diagnosed in boys than in girls. It is postulated that ASD is caused by a complex interaction between genetic and environmental factors. Epigenetics provides a mechanistic link between exposure to an unbalanced maternal diet and persistent modifications in gene expression levels that can lead to phenotype changes in the offspring. To better understand the impact of the early development environment on the risk of ASD in offspring, we assessed the effect of maternal high-fat (HFD), high-carbohydrate, and mixed diets on molecular changes in adolescent and young adult offspring frontal cortex and hippocampus. Our results showed that maternal HFD significantly altered the expression of 48 ASD-related genes in the frontal cortex of male offspring. Moreover, exposure to maternal HFD led to sex- and age-dependent changes in the protein levels of ANKRD11, EIF4E, NF1, SETD1B, SHANK1 and TAOK2, as well as differences in DNA methylation levels in the frontal cortex and hippocampus of the offspring. Taken together, it was concluded that a maternal HFD during pregnancy and lactation periods can lead to abnormal brain development within the transcription and translation of ASD-related genes mainly in male offspring.

## 1. Introduction

Autism spectrum disorder (ASD) is a complex neurodevelopmental disorder whose prevalence has increased rapidly over the last few decades [1]. One of the most characteristic symptoms of this heterogeneous brain disease is a significant disturbance of social interaction, communication, emotional circuits, and restricted and repetitive behavior patterns [2]. ASD is diagnosed more often in boys than in girls; the ratio is three-to-one male to female [3]. It is now well known that the development of ASD is caused by a complex interaction between genetic (40–80%) and environmental factors (most likely the remaining part of the risk), which are mainly related to epigenetic mechanisms [4]. Nongenetic factors can act independently or by interaction with ASD-associated gene mutations [5]. Among numerous environmental factors, such as the older age of parents, maternal infections, medications (valproate, antidepressants), smoking and alcohol use, imbalanced macro- and micronutrients, exposure to air pollution, heavy metals, and pesticides [6], maternal overweight or obesity increases the risk of ASD development in offspring by as much as 36% [7]. Moreover, recent considerations of the pathogenesis of ASD mainly emphasized the intrauterine and early postpartum development periods, characterizing ASD as a multistage, progressive disorder of brain development [8,9].

Clinical and preclinical data indicate that the period of intrauterine development and early childhood is extremely sensitive to external factors, such as an improper diet consumed by mothers, which may contribute to the occurrence of structural and functional abnormalities in the brain, predisposing offspring to numerous neurodevelopmental and mental diseases later in life [10]. The continuous increase in the incidence of ASD in the last two decades has intensified research on the role of nongenetic etiological factors, such as epigenetics (mainly abnormal DNA methylation) and environmental interactions [11,12,13].

Epigenetic changes occur throughout the entire life of an individual, especially during the period of intrauterine development. DNA methylation is a dynamic process that occurs mainly within the CpG dinucleotides common in the promoter regions of genes. Most often, high levels of methylation in the promoter regions are associated with decreased gene expression [14,15]. DNA methylation is essential for cell-specific gene expression and plays an important role during embryonic development and the early postnatal period, which corresponds to the peak time of synaptogenesis [16]. Numerous studies indicate that a maternal HFD alters the methylation pattern in the offspring [10,17,18,19]. As with epigenome reprogramming during early embryonic development, dynamic epigenetic remodeling in the postnatal brain may be a key stage for epimutation accumulation and subsequent dysregulation of brain development and function. In addition, the epigenetic hypothesis may also take into account environmental factors in molecular pathogenesis, as it has been well documented that 5-methylcytosine (5-mC) can be modified by environmental factors both in the prenatal and postnatal periods [12].

To better understand the role of an improperly balanced maternal diet on the risk of ASD in offspring exposed during pregnancy and lactation, in this study, we assessed the effects of a maternal high-fat diet (HFD), a high-carbohydrate diet (HCD; rich in sucrose), and a mixed diet (MD; rich in carbohydrate and fat) on expression disturbances in genes and proteins, the functioning of which is associated with the development of ASD symptoms. Next, we assessed the level of global DNA methylation as well as the changes in CpG island methylation in two brain regions, the frontal cortex and hippocampus, which are the structures involved in the pathology of this disorder.

## 2. Materials and Methods

### 2.1. Animals and Diets

All procedures were performed following EU Directive 2010/63/EU with the approval of the Ethical Committee at the Maj Institute of Pharmacology Polish Academy of Sciences (approval numbers 1270/2015 and 42/2017).

Wistar Han rats from Charles River (Germany) were housed in standard cages in an animal colony room maintained at 22 ± 2 °C and 55 ± 10% humidity under a 12 h light–dark cycle (lights on at 6:00 a.m.) with free access to water and food. Female virgin rats (200–240 g), after the acclimatization period and during the proestrus phase (smears from females were assessed to determine the estrous cycle phase), were mated with males.

The pregnancy was confirmed by examining vaginal smears for the presence of sperm. Then, pregnant females were individually housed and randomly assigned to one of the four groups: standard diet (SD; 65% carbohydrate, 13% fat, 22% protein, 3.4 kcal/g; VRF1; Special Diets Services, Essex, UK) or modified diets purchased from Altromin (Lage, Germany): high-fat diet (HFD; 24% carbohydrate, 60% fat, 16% protein, 5.31 kcal/g; C1057 mod.), high-carbohydrate diet (HCD; 70% carbohydrate: rich in sucrose—40%, 12% fat, 18% protein, 3.77 kcal/g; C1010) or mixed diet (MD; 56% carbohydrate, 28% fat, 16% protein, 3.90 kcal/g; C1011). Dams were fed these diets ad libitum during pregnancy (21 days) and lactation (21 days). The modified maternal diets used in these experiments did not affect the litter size or birth weight of offspring. Moreover, HFD-, HCD-, and MD-fed dams during pregnancy and lactation did not have significant differences in body weight compared to dams from the SD group (except during the last week of lactation, when the body weight of the MD dams was lower) [20]. Litter sizes were normalized to 10–12 pups with a sex ratio as close to 1:1 as possible. After weaning, offspring at postnatal day (PND) 22 were separated according to sex, housed 5 per cage, and switched to SD. Male and female offspring were used in this study.

### 2.2. Brain Tissue Collection

The subsets of offspring were sacrificed through rapid decapitation at PND 28 (adolescence) and PND 63 (early adulthood). The frontal cortex and hippocampus were dissected according to The Rat Brain Atlas [21]. The structures were immediately frozen on dry ice and stored at −80 °C until further molecular analysis. Animals were not fasting before decapitation. All samples were collected between 9:00 and 12:00 a.m.

### 2.3. RNA Sequencing

Frontal cortex RNA isolation (from offspring at PND 28), sequencing, data preprocessing, and Gene Ontology and KEGG pathway analysis results were described previously [22]. Raw data for this project are accessible from the Sequence Read Archive (SRA) under the PRJNA669556 BioProject. Briefly, gene-level transcript counts were quantified with the Cufflinks v 2.2.1 package. Two-way analysis of variance (ANOVA) was run on all genes with mean (log_2_(FPKM + 1)) > 1. The FDR (false discovery rate) was computed from two-way ANOVA p values. Next, genes with a mean (log_2_(FPKM + 1)) > 1 were filtered to include only transcripts from genes indexed in the SFARI database [23] as either human or animal model genes (n = 960 genes). Transcripts from 852 genes were detected. These were further filtered for FDR of the diet factor < 0.1 and FDR of the diet × sex interaction < 0.1. An additional filter of SD/mean < 0.3 in each group was applied to exclude genes with high within-group variability. This filtering yielded 48 genes. Pathway and Gene Ontology (GO) analysis was conducted with EnrichR [24,25]. Full results are available under this link: https://maayanlab.cloud/Enrichr/enrich?dataset=f4105d0ce5a9cec011c49a919df36efe (accessed on 9 September 2021).

### 2.4. Enzyme-Linked Immunosorbent Assay (ELISA)

The frontal cortex and hippocampus were homogenized (Bioprep-24 Homogenizer; Aosheng, Hangzhou, China) in ice-cold phosphate-buffered saline (PBS) buffer, pH 7.4 (Takara Bio, Göteborg, Sweden), with a protease inhibitor cocktail (Complete, Roche, Mannheim, Germany). The protein levels of Ankyrin Repeat Domain-Containing Protein 11 (ANKRD11; Cat# E2515Ra), Eukaryotic Translation Initiation Factor 4E (EIF4E; Cat# E2608Ra), Neurofibromin 1 (NF1; Cat# E2518Ra), Histone-Lysine N-methyltransferase SETD1B (SETD1B; Cat# E2517Ra), SH3 And Multiple Ankyrin Repeat Domains Protein 1 (SHANK1; Cat# E2516Ra) and Serine/Threonine-Protein Kinase TAOK2 (TAOK2; Cat# E2606Ra) were measured using ELISA kits (Bioassay Technology Laboratory, Shanghai, China) following the manufacturer’s protocol. Duplicates of each sample and series of standards were transferred to ELISA plates. Absorbance was measured at a wavelength of λ = 450 nm using a Multiskan Spectrum spectrophotometer (Thermo LabSystems, Philadelphia, PA, USA). The concentration of proteins was calculated from a standard curve and expressed as pg/mg of protein. For protein measurement, the Pierce BCA Protein Assay Kit (Thermo Scientific, Rockford, IL, USA) was used.

### 2.5. Quantifying Global DNA Methylation

DNA was isolated following the manufacturer’s protocol using the AllPrep DNA/RNA/Protein Mini Kit (Qiagen, Hilden, Germany). One hundred nanograms of DNA were used for the study. The level of global DNA methylation (5-mC%) was measured using a MethylFlash Global DNA Methylation (5-mC) ELISA Easy Kit (Cat# P-1030-96; Epigentek, Farmingdale, NY, USA) according to the manufacturer’s instructions. The absolute quantification of 5-mC% contents was performed using a standard curve according to the manufacturer’s manual. All samples were analyzed in duplicate.

### 2.6. EpiTect Methyl II PCR Assay

DNA was isolated following the manufacturer’s protocol using the AllPrep DNA/RNA/Protein Mini Kit (Qiagen, Germany). The purity and concentration of the extracted DNA were detected before further investigation using an ND-1000 spectrometer (NanoDrop Technologies Inc., Wilmington, DE, USA) and then stored at −20 °C until further restriction digestion. DNA methylation was detected using EpiTect Methyl II PCR Assays (Qiagen, Germantown, MD, USA). The technique is based on the detection of the remaining input genome after digestion with a methylation-sensitive restriction enzyme. First, digested samples of DNA were obtained following the manufacturer’s instructions for the EpiTect Methyl II DNA Restriction Kit (Qiagen, USA). Briefly, as follows: input genomic DNA was divided into four equal aliquots and tested with four tubes: no enzyme (Mo), methylation-sensitive enzyme (Ms), methylation-dependent enzyme (Md), and double enzymes (Msd). All 4 reactions were incubated at 37 °C overnight, followed by 65 °C for 20 min using a thermocycler (Applied Biosystems, Foster City, CA, USA). Next, the methylation status was determined using QuantStudio 3 (Applied Biosystems, USA), the enzymatic reactions were mixed directly with the qPCR master mix (RT2 SYBR Green ROX qPCR Mastermix (Qiagen, USA) and the plate containing the prealiquots was dispensed into the PCR primer mix (EpiTect Methyl II qPCR Primer Assay; Taok2 (Cat# EPRN100699–1A); Eif4e (Cat# EPRN104934–1A; Qiagen, USA)). Real-time PCR was performed under defined cycle conditions: 95 °C for 10 min (1 cycle), then 99 °C for 30 s and 72 °C for 1 min (3 cycles), and finally 97 °C for 15 s and 72 °C for 1 min (40 cycles). The results were obtained using a data analysis sheet (EpiTect Methyl II PCR Array data analysis template) that automatically calculates the relative amount of methylated and unmethylated DNA fractions.

### 2.7. Statistical Analysis 

All data are expressed as the mean ± standard error of the mean (SEM). Data were analyzed with two-way ANOVA (diet × sex) followed by a Bonferroni post hoc test using GraphPad Prism 9.1.0 software (GraphPad Software, La Jolla, CA, USA). *p* < 0.05 was considered statistically significant.

## 3. Results

### 3.1. Maternal HFD during Pregnancy and Lactation Changes the Expression of ASD-Related Genes in the Offspring Frontal Cortex

**Figure 1:**
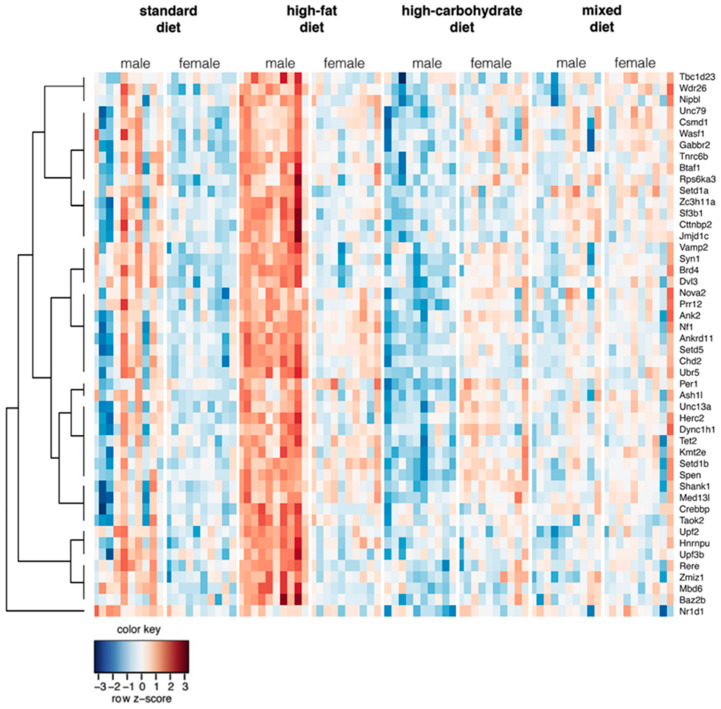
Maternal diet influences the differential expression of genes related to ASD in the offspring frontal cortex at PND 28. Heatmap of the gene expression of 48 selected DEGs. Gene expression values obtained by using RNA-seq were normalized (z-score).

We investigated whether genes reported to be related to ASD were differentially regulated in response to maternal dietary patterns. To this end, we filtered gene expression analysis results to include only genes reported in the SFARI database [23]. Furthermore, we focused on genes that are differentially regulated by maternal diets (FDR < 10%) and have significant interaction between sex and diet (FDR < 10%). We identified 48 ASD-related genes whose expression was significantly altered in adolescent (at PND 28) offspring in the frontal cortex after exposure to maternal HFD (Figure 1). For the HFD group as compared with the control animals, 47 genes were upregulated in males (average log_2_ of fold change 0.4) and 47 genes were upregulated in females (average log_2_ of fold change 0.17). For offspring from MD groups, these numbers were 16 and 46, and for HCD groups, these numbers were two and 45. Detailed results can be found in Supplementary Table S1. Pathway analysis of selected 48 genes showed the overrepresentation of the histone modification pathway in this gene set (WikiPathways database, term: Histone Modifications WP2369, 5/48 genes, adjusted *p* value: 0.00004). Top GO Molecular Function term was histone-lysine N-methyltransferase activity (GO:0018024) (5/48 genes, adjusted *p* value 0.000008). Moreover, the top 75 genes selected from the analysis of all protein coding genes without filtering [22] included five genes related to ASD: *Kif5c*, *Syn1*, *Sparcl1*, *Agap2*, and *Actb* (reported in the SFARI database).

### 3.2. Maternal HFD during Pregnancy and Lactation Changes the Levels of ASD-Related Proteins in the Offspring Brain

We chose for further analysis five functionally different proteins (ANKRD11, NF1, SETD1B, SHANK1, and TAOK2) encoded by genes whose expression changed after exposure to maternal HFD in the offspring frontal cortex and which have been assessed in our previous studies in the prefrontal cortex [26]. Additionally, we analyzed the level of the EIF4E protein responsible for translation initiation, the activity of which may be indirectly regulated by NF1 [27]. We also assessed protein levels and epigenetic changes in another important brain structure in the pathogenesis of ASD, the hippocampus, to assess whether the changes caused by maternal HFD are brain region specific.

**Figure 2:**
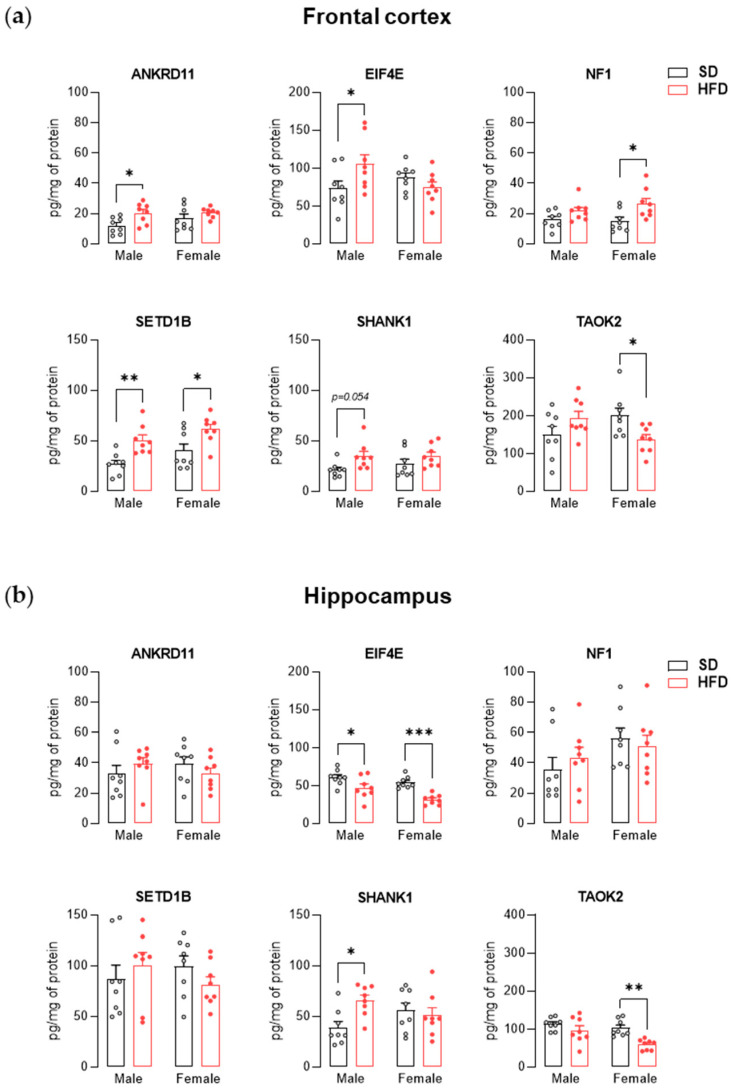
A maternal high-fat diet (HFD) during pregnancy and lactation changes the level of proteins associated with autism spectrum disorder (ASD) in the frontal cortex and hippocampus of adolescent offspring. (**a**) Effects of maternal HFD on ANKRD11, EIF4E, NF1, SETD1B, SHANK1, and TAOK2 protein levels in the frontal cortex of male and female offspring at PND 28. (**b**) Effects of maternal HFD on ANKRD11, EIF4E, NF1, SETD1B, SHANK1, and TAOK2 protein levels in the hippocampus of male and female offspring at PND 28. The results are expressed as the mean (±SEM). *n* = 8 rats/group. Data were analyzed by two-way ANOVA followed by Bonferroni post hoc test. * *p* < 0.05, ** *p* < 0.01, *** *p* < 0.001 versus the standard diet (SD) group.

Two-way ANOVA showed a significant effect of maternal diet on the level of ANKRD11 (F_(1,28)_ = 7.74, *p* < 0.01), NF1 (F_(1,28)_ = 10.43, *p* < 0.01), SETD1B (F_(1,28)_ = 18.61, *p* < 0.001) and SHANK1 (F_(1,28)_ = 6.82, *p* < 0.05), as well as effect of diet × sex interaction on the level of EIF4E (F_(1,28)_ = 5.93, *p* < 0.05) and TAOK2 (F_(1,28)_ = 8.48, *p* < 0.01) in the offspring frontal cortex at PND 28. After exposure to a maternal HFD, there was a significant increase in the level of ANKRD11 (*p* < 0.05), EIF4E (*p* < 0.05), and SETD1B (*p* < 0.01) as well as an upward trend for the SHANK1 protein (*p* = 0.054) (Figure 2a) in male offspring compared to the control group. In adolescent females from the HFD group, an increase in the levels of NF1 protein (*p* < 0.05) and SETD1B (*p* < 0.05) was observed, with a simultaneous decrease in the level of TAOK2 protein (*p* < 0.05) compared to the SD group (Figure 2a). Within the hippocampus, two-way ANOVA showed a significant effect of maternal diet on the level of EIF4E (F_(1,28)_ = 25.72, *p* < 0.001) and TAOK2 (F_(1,28)_ = 13.81, *p* < 0.001) or effect of diet × sex interaction on the level of SHANK1 (F_(1,28)_ = 5.71, *p* < 0.05). Post hoc analysis showed that exposure to maternal HFD increased the hippocampal level of SHANK1 (*p* < 0.05) in males and decreased TAOK2 level (*p* < 0.01) in female offspring compared to the control animals at PND 28. Additionally, regardless of the sex of the offspring, a decrease in EIF4E was noted in the HFD group (male *p* < 0.05; female *p* < 0.001) compared to the SD group at PND 28 (Figure 2b).

**Figure 3:**
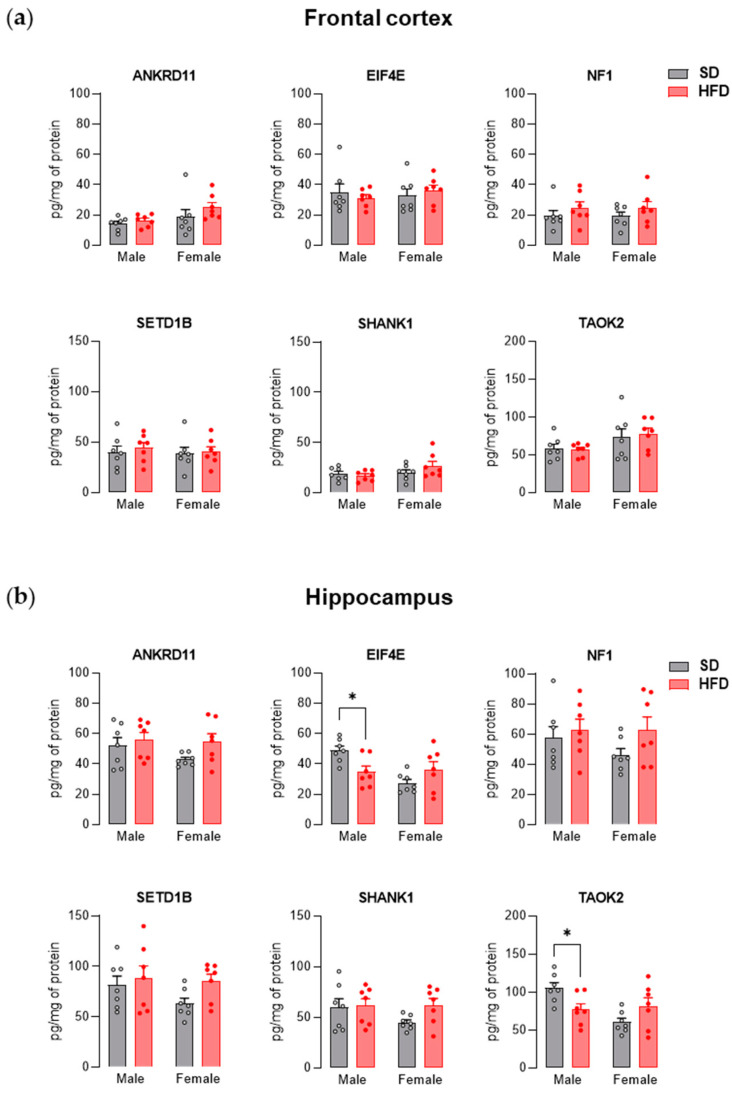
A maternal high-fat diet (HFD) during pregnancy and lactation changes the level of proteins associated with autism spectrum disorder (ASD) in the hippocampus of young adult offspring. (**a**) Effects of maternal HFD on ANKRD11, EIF4E, NF1, SETD1B, SHANK1, and TAOK2 protein levels in the frontal cortex of male and female offspring at PND 63. (**b**) Effects of maternal HFD on ANKRD11, EIF4E, NF1, SETD1B, SHANK1, and TAOK2 protein levels in the hippocampus of male and female offspring at PND 63. The results are expressed as the mean (±SEM). *n* = 7 rats/group. Data were analyzed by two-way ANOVA followed by Bonferroni post hoc test. * *p* < 0.05 versus the standard diet (SD) group.

During early adulthood (PND 63), the levels of the tested proteins in the frontal cortex did not differ significantly between the offspring from the control group and the HFD group (Figure 3a). Two-way ANOVA showed a significant effect of diet × sex interaction on the hippocampal levels of EIF4E (F_(1,24)_ = 9.32, *p* < 0.01) and TAOK2 (F_(1,24)_ = 9.49, *p* < 0.01). In the hippocampus, a decrease in the levels of EIF4E (*p* < 0.05) and TAOK2 (*p* < 0.05) proteins was noted only in the case of male offspring exposed to maternal HFD (Figure 3b). A summary of the influence of maternal HFD on the level of tested proteins is presented in Table 1.

### 3.3. Maternal HFD during Pregnancy and Lactation Changes Global DNA metHylation in the Offspring Brain

**Figure 4:**
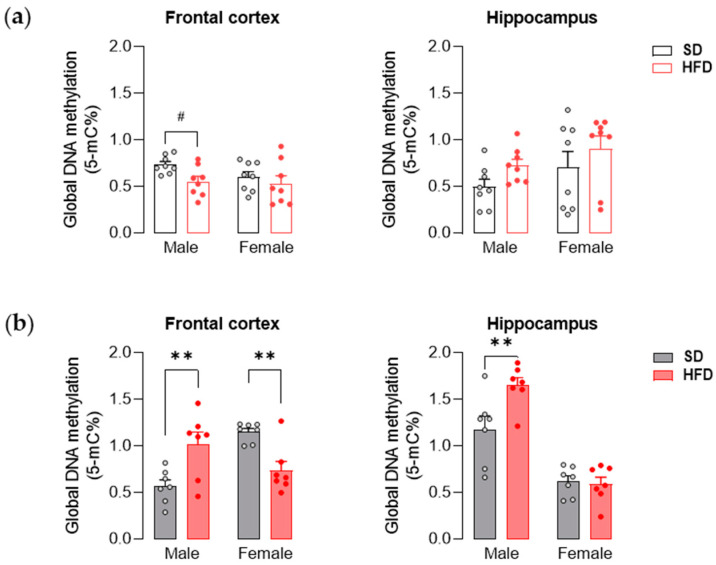
A maternal high-fat diet (HFD) during pregnancy and lactation changes global DNA methylation in the frontal cortex and hippocampus of adolescent and young adult offspring. (**a**) Effects of maternal HFD on global DNA methylation in the frontal cortex and hippocampus of male and female offspring at PND 28. (**b**) Effects of maternal HFD on global DNA methylation in the frontal cortex and hippocampus of male and female offspring at PND 63. The results are expressed as the mean (±SEM). *n* = 8 rats/group (PND 28); *n* = 7 rats/group (PND 63). Data were analyzed by two-way ANOVA followed by Bonferroni post hoc test. ^#^ *p* = 0.08, ** *p* < 0.01 versus the standard diet (SD) group.

During adolescence, in offspring, two-way ANOVA showed a significant effect of maternal diet on global DNA methylation (F_(1,28)_ = 4.52, *p* < 0.05) in the frontal cortex. However, post hoc analysis indicated that only the male offspring of the HFD group showed a trend toward lower global DNA methylation (*p* = 0.08) compared to the control group. At the same time, exposure to maternal HFD did not affect hippocampal global DNA methylation at PND 28 (Figure 4a).

At PND 63 statistical analysis showed a significant effect of maternal diet on global DNA methylation within the hippocampus (F_(1,24)_ = 5.48, *p* < 0.05) and effect of diet × sex interaction in the frontal cortex (F_(1,24)_ = 22.67, *p* < 0.001) and hippocampus (F_(1,24)_ = 7.06, *p* < 0.05). In adult male offspring from the HFD group, the methylation level was increased in the frontal cortex and hippocampus (*p* < 0.01). On the other hand, decreased levels of global DNA methylation were observed in the frontal cortex of female offspring exposed to maternal HFD (*p* < 0.01) (Figure 4b).

### 3.4. Maternal HFD during Pregnancy and Lactation Alters CpG Island DNA Methylation in the Offspring Brain

**Figure 5:**
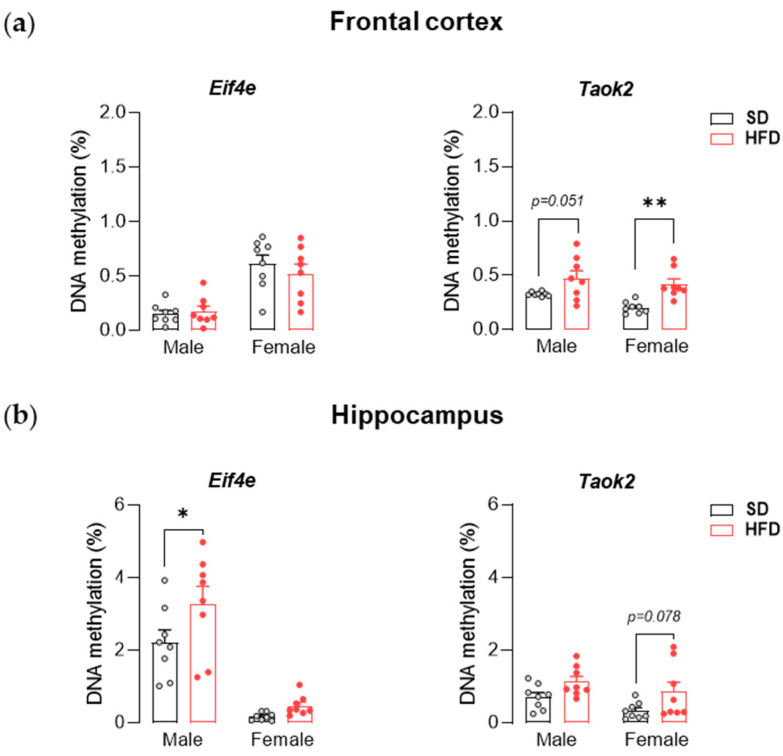
A maternal high-fat diet (HFD) during pregnancy and lactation changes the DNA methylation of CpG island of *Eif4e* and *Taok2* in the frontal cortex and hippocampus of adolescent offspring. (**a**) Effects of maternal HFD on DNA methylation of CpG island of *Eif4e* and *Taok2* in the frontal cortex of male and female offspring at PND 28. (**b**) Effects of maternal HFD on DNA methylation of CpG island of *Eif4e* and *Taok2* in the hippocampus of male and female offspring at PND 28. The results are expressed as the mean (±SEM). n = 8 rats/group. Data were analyzed by two-way ANOVA followed by Bonferroni post hoc test. * *p* < 0.05, ** *p* < 0.01 versus the standard diet (SD) group.

For more detailed information on the effects of maternal diet during pregnancy and lactation on epigenetic changes related to DNA methylation, we assessed the CpG island methylation of *Eif4e* and *Taok2* within the frontal cortex and hippocampus in juvenile offspring. Two-way ANOVA showed a significant effect of maternal diet on the DNA methylation of CpG island of *Taok2* in the frontal cortex (F_(1,28)_ = 17.49, *p* < 0.001) and hippocampus (F_(1,28)_ = 7.67, *p* < 0.01) as well as *Eif4e* within the hippocampus (F_(1,28)_ = 5.15, *p* < 0.05). We observed the hypermethylation of CpG islands of *Taok2* in the frontal cortex of male (*p* = 0.051) and female (*p* < 0.01) offspring exposed to maternal HFD compared to control animals (Figure 5a). In addition, in the hippocampus, an increase in methylation of CpG islands of *Eif4e* was observed in male offspring from the HFD group (*p* < 0.05), and an upward trend was observed in female offspring in *Taok2* (*p* = 0.078) (Figure 5b).

## 4. Discussion

Growing epidemiological and preclinical evidence highlights the crucial role of the maternal diet during pregnancy and lactation in the normal development of the brain and the risk of nervous system diseases in offspring [10,20,26,28,29]. Our recent studies have indicated that maternal HFD during pregnancy and the suckling period leads to the disturbance of social interactions and an increase in repetitive behavior (assessed in the marble-burying and self-grooming test) in male offspring during the adolescent period compared to control animals [26]. Behavioral changes observed in the offspring suggest that exposure to maternal HFD early in life may increase the risk of developing an autistic phenotype. A reduction in social interactions and an increase in repetitive behavior are characteristic of numerous preclinical ASD models (e.g., mice lacking ASD-related genes, BTBR mice, offspring exposure prenatally to VPA) [30]. Therefore, we decided to look for molecular changes that could account for the behavioral problems of the offspring in two brain structures, the frontal cortex and the hippocampus, which are responsible for the control of numerous functions that are disturbed in ASD patients [31,32,33]. The frontal cortex is responsible for controlling important executive functions of the brain, such as social behavior, communication, emotions, and planning and decision-making [31]. In turn, the hippocampus is involved in cognitive processes related to learning and memory, spatial navigation, and emotional behaviors [34]. Clinical studies confirm that disturbances in the cortical growth pattern, changes in the morphology and thickness of the cortex, or disorganization of cortical neurons are observed in ASD patients [31] as well as changes in the size and density of neurons within the hippocampus [32].

The present research confirms that a maternal HFD during pregnancy and lactation generates significant molecular changes in the examined structures in the offspring throughout their lives. Our transcriptome profiling results in the frontal cortex indicated that males exposed to maternal HFD at PND 28 showed significant changes in the expression of ASD-related genes. Therefore, these results are consistent with previous observations in which exposure to maternal HFD generated autistic-like behaviors in male, but not female, offspring during adolescence [26]. The observed sex differences in gene expression were confirmed by previous studies in which HFD-induced maternal obesity resulted in a significantly greater change in the gene expression profile in male versus female mouse fetal brains. The factors that may influence the sex-related influence of maternal HFD on offspring brain development include differences in exposure to androgens, the increased susceptibility of the male fetus to inflammation in utero, and the different activities of astrocytes and microglia [35]. Interestingly, the presence of a greater number of compensatory mechanisms and molecular, structural, and hormonal differences compared to males, called female protective effects, can protect against ASD development in females [36,37,38]. Similar changes were not observed in subjects exposed to maternal HCD and MD, which indicates the specificity of the observed changes depending on the type of modification in the maternal diet and a high-fat intrauterine development environment as a predisposing factor to an ASD-like phenotype. Gene set enrichment analysis revealed that the selected genes were likely to be involved in histone modifications and thus regulate chromatin organization and gene expression. Among the 48 genes with disturbed expression by maternal HFD expression, there are genes encoding proteins involved in three pathways crucial for the development of ASD (synaptic function, chromatin remodeling, transcription regulation) [39,40,41]. At PND 28 in the frontal cortex of male offspring from the HFD group, an increase in the expression of, inter alia, genes was observed, including *Cttnbp2*, *Gabbr2*, *Nf1*, *Shank1*, *Syn1*, *Taok2* (involved in synaptic function [42,43,44,45,46,47]), *Ankrd11*, *Ash1l*, *Baz2b*, *Crebbp*, *Setd1b*, *Kmt2e* (involved in chromatin regulation [48,49,50,51,52,53]), *Ankrd11*, *Btaf1*, *Med13l*, *Nf1*, *Spen*, and *Taok2* (involved in transcription regulation [49,54,55,56,57,58]). A majority of high-confidence ASD genes, including genes whose proper expression was disturbed in the frontal cortex of male offspring exposed to maternal HFD, are characterized by functional pleiotropy and play a key role in proper brain development and function [8]. The direction and lifetime point when pathways related to genes with pleiotropic functions are dysregulated will lead to different, even opposing, effects, resulting in neural and symptomatic heterogeneity reported in individuals with ASD [8]. Moreover, the simultaneous decreased or increased expression of numerous genes is noted in different brain regions from ASD transcriptomic study patients, and for a set of genes, the opposite direction of changes was observed, depending on the study (see review [59]). The expression profile of ASD-related genes is also subject to changes at different stages of life [60].

In the frontal cortex and hippocampus of adolescent and adult offspring from the HFD group, we also assessed the levels of several proteins with various functions encoded by genes altered by maternal diet. At PND 28, male offspring exposed to maternal HFD showed an increased level of ANKRD11, SETD1B, SHANK1, and EIF4E proteins in the frontal cortex and SHANK1 and EIF4E in the hippocampus compared to control animals. On the other hand, in females, increased cortical concentrations of NF1 and SETD1B and decreased levels of EIF4E in the hippocampus and TAOK2 in the frontal cortex and hippocampus were noted. ANKRD11 is responsible for regulating transcription by interacting with chromatin-modifying enzymes (histone deacetylases and histone acetyltransferases) and is involved in the proper development of the brain, neuronal differentiation through the BDNF/TrkB signaling pathway, and synaptic plasticity [49,61]. Similar to ANKRD11, SETD1B also participates in the epigenetic regulation of gene expression by modifying the chromatin structure related to histone methylation [51,62]. TAOK2 is involved in mitogen-activated protein (MAP) kinase pathways that can modulate gene transcription [63] and play a part in dendrite and dendritic spine development [55,64]. In turn, NF1 is a negative regulator of RAS signaling, through which it can regulate mTOR or MEK/MAPK cascades, which are key regulators of synaptogenesis and protein synthesis (among others, with the participation of the translational factor EIF4E) [65,66,67]. Disorders within the mTOR and MAPK pathways are characteristic of patients with ASD (e.g., an increase in protein p-EIF4E, rpS6, ERK1–2 in blood) and are an important target in the search for a treatment for this brain disease [68,69]. Moreover, in preclinical studies, the overexpression of EIF4E in microglia results in autistic-like behavior in male (but not female) mice as well as an increased spine density and excitation-to-inhibition ratio within cortical neurons [70]. The observations of Xu et al. [70] may be relevant to a better understanding of the autistic-like phenotype in male offspring exposed to maternal HFD during pregnancy and lactation. An increased amount of cortical protein EIF4E was found in the frontal cortex of juvenile male offspring from the HFD group, who also had an increased concentration of SHANK1 (scaffolding proteins of excitatory synapses, associated with the structural and functional organization of the dendritic spine), which may suggest increased spine density in these animals [71,72]. Increased cortical spine density has also been reported in some ASD patients [73,74]. Additionally, in our previous work, we showed a reduction in social interactions [26] and disturbances in the expression of neuronal markers specific to excitatory and inhibitory cortical neurons (upregulated markers of excitatory neurons, while at the same time downregulated markers of inhibitory neurons) [22] in adolescent offspring exposed to maternal HFD. To confirm these assumptions, specific studies are needed because in the present study, the level of the EIF4E protein was determined in the homogenate of the frontal cortex, which does not allow for the comparison of its amount within the microglia. Changing the diet to fully balanced after weaning contributed to the reduction of the differences in the level of examined proteins in the frontal cortex assessed in adult animals. In animals from the HFD group at PND 63, only a reduction in hippocampal concentration was observed for TAOK2 and EIF4E. Molecular results appear to be consistent with behavioral observations in which disturbances in social interactions and an increase in repetitive behavior are seen in juvenile, but not adult, offspring exposed to maternal HFD [26].

One of the major epigenetic mechanisms by which the prenatal development environment (including maternal HFD) may significantly influence the phenotype later in life is DNA methylation [13,17,75]. In our study, we observed a downward trend in the level of global DNA methylation in the frontal cortex in male offspring from the HFD group at PND 28. Male offspring-specific decreased levels of cortical DNA methylation potentially explain the increased expression of ASD-related genes noted in the present study, as DNA methylation in promoters or gene regulatory regions inhibit transcription [18]. Interestingly, in adult males exposed to maternal HFD, an increase in global DNA methylation was noted in both structures studied. At the same time, a significant decrease in the level of DNA methylation was observed in adult females in the frontal cortex. Together, these results indicate that maternal HFD during pregnancy and lactation significantly influences DNA methylation patterns in the offspring’s brain, which may be manifested by disturbances in the expression of numerous genes. It is worth noting that in the cortical structures of individuals with ASD, significant differences in the level of DNA methylation were observed compared to the control groups [76].

Herein, to confirm the impact of epigenetic changes on the expression of specific genes, we investigated the DNA methylation of CpG islands of two selected genes (*Eif4e* and *Taok2*), whose transcription and translation were impaired upon exposure to maternal HFD. In the frontal cortex, in adolescent females exposed to maternal HFD, an upward trend of DNA methylation of *Taok2* was observed in males. Interestingly, despite the increase in DNA methylation, *Taok2* expression was increased in males and did not differ in females exposed to maternal HFD compared to control animals. In turn, females from the HFD group showed a tendency to increase the DNA methylation of *Taok2* within the hippocampus while simultaneously reducing the level of TAOK2 protein. Additionally, the increasing DNA methylation of *Eif4e* in the hippocampus in males at PND 28 translates into a reduction in the level of the protein encoded by this gene (although a decrease in concentration was also observed in females, despite the lack of differences in the level of DNA methylation). The above results indicate that maternal HFD can significantly change the level of CpG island methylation in offspring, which can only be explained by some of the noted changes in the brain. It is worth emphasizing that there are other epigenetic mechanisms, such as histone modifications and noncoding RNAs, that can also mediate gene regulation [77] and were not assessed in this study.

In summary, the presented results indicate a key role of maternal HFD during pregnancy and lactation in the disturbance of normal brain development, which may predispose offspring to the development of ASD symptoms. Exposure to a maternal HFD alters the transcription and translation of ASD-related genes mainly in male offspring, and these changes may result from epigenetic modifications (including the degree of CpG island methylation). It is worth emphasizing the heterogeneity of the observed molecular changes depending on the brain region, timeline, and sex of the offspring, which is also observed in the pathomechanisms and symptomatology of ASD in humans. Thus, further research on the role of the maternal diet in the risk of developing ASD is needed.

## Figures and Tables

**Table 1:** Summary of the effects of a maternal HFD during pregnancy and lactation on ASD-related protein levels in the offspring brain.

	Frontal Cortex				Hippocampus			
	Males		Females		Males		Females	
	PND 28	PND 63	PND 28	PND 63	PND 28	PND 63	PND 28	PND 63
ANKRD11	↑	−	−	−	−	−	−	−
EIF4E	↑	−	−	−	↓	↓	↓	−
NF1	−	−	↑	−	−	−	−	−
SETD1B	↑	−	↑	−	−	−	−	−
SHANK1	↑ ^#^	−	−	−	↑	−	−	−
TAOK2	−	−	↓	−	−	↓	↓	−

Symbols: ↑: increased; ↓: decreased; −: unchanged; ^#^: tendency.

## Data Availability

RNA-seq raw data are deposited in the SRA database under the PRJNA669556 BioProject.

## Supplementary Materials

The following are available online at https://www.mdpi.com/article/10.3390/nu13093212/s1, Table S1: RNA-seq analysis results for 48 selected ASD-related genes. Sheet1 (ANOVA_sds_mean): Columns fdr.sex, fdr.diet and fdr.interaction show two-way ANOVA results. Two-way ANOVA was conducted on all genes. Further columns show SD and mean values for each gene in each group. Sheet 2 (regulation)-fold change columns: log_2_(fold change) for each of the selected genes for each diet provided for males and females, regulation columns: direction of gene expression change as compared with SD for each sex. FPKM data for all genes are available in the Supplementary Material of Gawlińska et al., 2021 [22]. Raw data are deposited in the SRA database under the PRJNA669556 BioProject.

## References

[1] Kogan M.D., Vladutiu C.J., Schieve L.A., Ghandour R.M., Blumberg S.J., Zablotsky B., Perrin J.M., Shattuck P., Kuhlthau K.A., Harwood R.L. (2018). The Prevalence of Parent-Reported Autism Spectrum Disorder among US Children. Pediatrics.

[2] Jonsson U., Alaie I., Löfgren Wilteus A., Zander E., Marschik P.B., Coghill D., Bölte S. (2017). Annual Research Review: Quality of life and childhood mental and behavioural disorders—A critical review of the research. J. Child Psychol. Psychiatry Allied Discip..

[3] Eggebrecht A.T., Dworetsky A., Hawks Z., Coalson R., Adeyemo B., Davis S., Gray D., McMichael A., Petersen S.E., Constantino J.N. (2020). Brain function distinguishes female carriers and non-carriers of familial risk for autism. Mol. Autism.

[4] Rylaarsdam L., Guemez-Gamboa A. (2019). Genetic Causes and Modifiers of Autism Spectrum Disorder. Front. Cell. Neurosci..

[5] Le Belle J.E., Sperry J., Ngo A., Ghochani Y., Laks D.R., López-Aranda M., Silva A.J., Kornblum H.I. (2014). Maternal inflammation contributes to brain overgrowth and autism-associated behaviors through altered redox signaling in stem and progenitor cells. Stem Cell Rep..

[6] Bölte S., Girdler S., Marschik P.B. (2019). The contribution of environmental exposure to the etiology of autism spectrum disorder. Cell. Mol. Life Sci..

[7] Sanchez C.E., Barry C., Sabhlok A., Russell K., Majors A., Kollins S.H., Fuemmeler B.F. (2018). Maternal pre-pregnancy obesity and child neurodevelopmental outcomes: A meta-analysis. Obes. Rev..

[8] Courchesne E., Pramparo T., Gazestani V.H., Lombardo M.V., Pierce K., Lewis N.E. (2019). The ASD Living Biology: From cell proliferation to clinical phenotype. Mol. Psychiatry.

[9] Courchesne E., Gazestani V.H., Lewis N.E. (2020). Prenatal Origins of ASD: The When, What, and How of ASD Development. Trends Neurosci..

[10] Gawlińska K., Gawliński D., Filip M., Przegaliński E. (2021). Relationship of maternal high-fat diet during pregnancy and lactation to offspring health. Nutr. Rev..

[11] Keil K.P., Lein P.J. (2016). DNA methylation: A mechanism linking environmental chemical exposures to risk of autism spectrum disorders?. Environ. Epigenetics.

[12] Tremblay M.W., Jiang Y.H. (2019). DNA methylation and susceptibility to autism spectrum disorder. Annu. Rev. Med..

[13] McKee S.E., Zhang S., Chen L., Rabinowitz J.D., Reyes T.M. (2018). Perinatal high fat diet and early life methyl donor supplementation alter one carbon metabolism and DNA methylation in the brain. J. Neurochem..

[14] Indrio F., Martini S., Francavilla R., Corvaglia L., Cristofori F., Mastrolia S.A., Neu J., Rautava S., Spena G.R., Raimondi F. (2017). Epigenetic matters: The link between early nutrition, microbiome, and long-term health development. Front. Pediatr..

[15] Franzago M., Fraticelli F., Stuppia L., Vitacolonna E. (2019). Nutrigenetics, epigenetics and gestational diabetes: Consequences in mother and child. Epigenetics.

[16] Bansal A., Simmons R.A. (2018). Epigenetics and developmental origins of diabetes: Correlation or causation?. Am. J. Physiol.-Endocrinol. Metab..

[17] Li Y. (2018). Epigenetic mechanisms link maternal diets and gut microbiome to obesity in the offspring. Front. Genet..

[18] Zhang Q., Xiao X., Zheng J., Li M., Yu M., Ping F., Wang T., Wang X. (2019). A Maternal High-Fat Diet Induces DNA Methylation Changes That Contribute to Glucose Intolerance in Offspring. Front. Endocrinol..

[19] Kong L., Chen X., Gissler M., Lavebratt C. (2020). Relationship of prenatal maternal obesity and diabetes to offspring neurodevelopmental and psychiatric disorders: A narrative review. Int. J. Obes..

[20] Gawliński D., Gawlińska K., Frankowska M., Filip M. (2020). Maternal Diet Influences the Reinstatement of Cocaine-Seeking Behavior and the Expression of Melanocortin-4 Receptors in Female Offspring of Rats. Nutrients.

[21] Paxinos G., Watson C. (1998). The Rat Brain in Stereotaxic Coordinates.

[22] Gawlińska K., Gawliński D., Korostyński M., Borczyk M., Frankowska M., Piechota M., Filip M., Przegaliński E. (2021). Maternal dietary patterns are associated with susceptibility to a depressive-like phenotype in rat offspring. Dev. Cogn. Neurosci..

[23] Abrahams B.S., Arking D.E., Campbell D.B., Mefford H.C., Morrow E.M., Weiss L.A., Menashe I., Wadkins T., Banerjee-Basu S., Packer A. (2013). SFARI Gene 2.0: A community-driven knowledgebase for the autism spectrum disorders (ASDs). Mol. Autism.

[24] Chen E.Y., Tan C.M., Kou Y., Duan Q., Wang Z., Meirelles G.V., Clark N.R., Ma’ayan A. (2013). Enrichr: Interactive and collaborative HTML5 gene list enrichment analysis tool. BMC Bioinform..

[25] Xie Z., Bailey A., Kuleshov M.V., Clarke D.J.B., Evangelista J.E., Jenkins S.L., Lachmann A., Wojciechowicz M.L., Kropiwnicki E., Jagodnik K.M. (2021). Gene Set Knowledge Discovery with Enrichr. Curr. Protoc..

[26] Gawlińska K., Gawliński D., Kowal-Wiśniewska E., Jarmuż-Szymczak M., Filip M. (2021). Alteration of the Early Development Environment by Maternal Diet and the Occurrence of Autistic-like Phenotypes in Rat Offspring. Int. J. Mol. Sci..

[27] Quesnel-Vallières M., Weatheritt R.J., Cordes S.P., Blencowe B.J. (2019). Autism spectrum disorder: Insights into convergent mechanisms from transcriptomics. Nat. Rev. Genet..

[28] Cheatham C.L. (2020). Nutritional Factors in Fetal and Infant Brain Development. Ann. Nutr. Metab..

[29] Gawliński D., Gawlińska K., Frankowska M., Filip M. (2020). Maternal high-sugar diet changes offspring vulnerability to reinstatement of cocaine-seeking behavior: Role of melanocortin-4 receptors. FASEB J..

[30] Ergaz Z., Weinstein-Fudim L., Ornoy A. (2016). Genetic and non-genetic animal models for autism spectrum disorders (ASD). Reprod. Toxicol..

[31] Donovan A.P.A., Basson M.A. (2017). The neuroanatomy of autism—A developmental perspective. J. Anat..

[32] Hashem S., Nisar S., Bhat A.A., Yadav S.K., Azeem M.W., Bagga P., Fakhro K., Reddy R., Frenneaux M.P., Haris M. (2020). Genetics of structural and functional brain changes in autism spectrum disorder. Transl. Psychiatry.

[33] Richards R., Greimel E., Kliemann D., Koerte I.K., Schulte-Körne G., Reuter M., Wachinger C. (2020). Increased hippocampal shape asymmetry and volumetric ventricular asymmetry in autism spectrum disorder. NeuroImage Clin..

[34] Strange B.A., Witter M.P., Lein E.S., Moser E.I. (2014). Functional organization of the hippocampal longitudinal axis. Nat. Rev. Neurosci..

[35] Edlow A.G., Guedj F., Pennings J.L.A., Sverdlov M.D., Neri C., Bianchi D.W. (2016). Males are from Mars, females are from Venus: Sex-specific fetal brain gene expression signatures in a mouse model of maternal diet-induced obesity. Am. J. Obstet. Gynecol..

[36] Robinson E.B., Lichtenstein P., Anckarsäter H., Happé F., Ronald A. (2013). Examining and interpreting the female protective effect against autistic behavior. Proc. Natl. Acad. Sci. USA.

[37] Werling D.M., Geschwind D.H. (2013). Sex differences in autism spectrum disorders. Curr. Opin. Neurol..

[38] Zhang Y., Li N., Li C., Zhang Z., Teng H., Wang Y., Zhao T., Shi L., Zhang K., Xia K. (2020). Genetic evidence of gender difference in autism spectrum disorder supports the female-protective effect. Transl. Psychiatry.

[39] De Rubeis S., He X., Goldberg A.P., Poultney C.S., Samocha K., Cicek A.E., Kou Y., Liu L., Fromer M., Walker S. (2014). Synaptic, transcriptional and chromatin genes disrupted in autism. Nature.

[40] Ayhan F., Konopka G. (2019). Regulatory genes and pathways disrupted in autism spectrum disorders. Prog. Neuro-Psychopharmacol. Biol. Psychiatry.

[41] Sullivan J.M., De Rubeis S., Schaefer A. (2019). Convergence of spectrums: Neuronal gene network states in autism spectrum disorder. Curr. Opin. Neurobiol..

[42] Lugarà E., De Fusco A., Lignani G., Benfenati F., Humeau Y. (2019). Synapsin I controls synaptic maturation of long-range projections in the lateral amygdala in a targeted selective fashion. Front. Cell. Neurosci..

[43] Shih P.Y., Hsieh B.Y., Lin M.H., Huang T.N., Tsai C.Y., Pong W.L., Lee S.P., Hsueh Y.P. (2020). CTTNBP2 Controls Synaptic Expression of Zinc-Related Autism-Associated Proteins and Regulates Synapse Formation and Autism-like Behaviors. Cell Rep..

[44] Fatemi S.H., Folsom T.D., Reutiman T.J., Thuras P.D. (2009). Expression of GABAB Receptors Is Altered in Brains of Subjects with Autism. Cerebellum.

[45] Richter M., Murtaza N., Scharrenberg R., White S.H., Johanns O., Walker S., Yuen R.K.C., Schwanke B., Bedürftig B., Henis M. (2019). Altered TAOK2 activity causes autism-related neurodevelopmental and cognitive abnormalities through RhoA signaling. Mol. Psychiatry.

[46] Shih Y.T., Huang T.N., Hu H.T., Yen T.L., Hsueh Y.P. (2020). Vcp Overexpression and Leucine Supplementation Increase Protein Synthesis and Improve Fear Memory and Social Interaction of Nf1 Mutant Mice. Cell Rep..

[47] Shi R., Redman P., Ghose D., Hwang H., Liu Y., Ren X., Ding L.J., Liu M., Jones K.J., Xu W. (2017). Shank Proteins Differentially Regulate Synaptic Transmission. Eneuro.

[48] Collins B.E., Greer C.B., Coleman B.C., Sweatt J.D. (2019). Histone H3 lysine K4 methylation and its role in learning and memory. Epigenetics Chromatin.

[49] Gallagher D., Voronova A., Zander M.A., Cancino G.I., Bramall A., Krause M.P., Abad C., Tekin M., Neilsen P.M., Callen D.F. (2015). Ankrd11 is a chromatin regulator involved in autism that is essential for neural development. Dev. Cell.

[50] Medrano-Fernández A., Delgado-Garcia J.M., del Blanco B., Llinares M., Sánchez-Campusano R., Olivares R., Gruart A., Barco A. (2019). The Epigenetic Factor CBP Is Required for the Differentiation and Function of Medial Ganglionic Eminence-Derived Interneurons. Mol. Neurobiol..

[51] Roston A., Evans D., Gill H., McKinnon M., Isidor B., Cogné B., Mwenifumbo J., Van Karnebeek C., An J., Jones S.J.M. (2021). SETD1B -associated neurodevelopmental disorder. J. Med. Genet..

[52] Scott T.M., Guo H., Eichler E.E., Rosenfeld J.A., Pang K., Liu Z., Lalani S., Bi W., Yang Y., Bacino C.A. (2020). BAZ2B haploinsufficiency as a cause of developmental delay, intellectual disability, and autism spectrum disorder. Hum. Mutat..

[53] Zhang C., Xu L., Zheng X., Liu S., Che F. (2021). Role of Ash1l in Tourette syndrome and other neurodevelopmental disorders. Dev. Neurobiol..

[54] Adegbola A., Musante L., Callewaert B., Maciel P., Hu H., Isidor B., Picker-Minh S., Le Caignec C., Chiaie B.D., Vanakker O. (2015). Redefining the MED13L syndrome. Eur. J. Hum. Genet..

[55] De Anda F.C., Rosario A.L., Durak O., Tran T., Gräff J., Meletis K., Rei D., Soda T., Madabhushi R., Ginty D.D. (2012). Autism spectrum disorder susceptibility gene TAOK2 affects basal dendrite formation in the neocortex. Nat. Neurosci..

[56] Klejman M.P., Zhao X., van Schaik F.M.A., Herr W., Timmers H.T.M. (2005). Mutational analysis of BTAF1-TBP interaction: BTAF1 can rescue DNA-binding defective TBP mutants. Nucleic Acids Res..

[57] Radio F.C., Pang K., Ciolfi A., Levy M.A., Hernández-García A., Pedace L., Pantaleoni F., Liu Z., de Boer E., Jackson A. (2021). SPEN haploinsufficiency causes a neurodevelopmental disorder overlapping proximal 1p36 deletion syndrome with an episignature of X chromosomes in females. Am. J. Hum. Genet..

[58] Yuen R.K.C., Merico D., Bookman M., Howe J.L., Thiruvahindrapuram B., Patel R.V., Whitney J., Deflaux N., Bingham J., Wang Z. (2017). Whole genome sequencing resource identifies 18 new candidate genes for autism spectrum disorder. Nat. Neurosci..

[59] Ansel A., Rosenzweig J.P., Zisman P.D., Melamed M., Gesundheit B. (2017). Variation in gene expression in autism spectrum disorders: An extensive review of transcriptomic studies. Front. Neurosci..

[60] Chow M.L., Pramparo T., Winn M.E., Barnes C.C., Li H.R., Weiss L., Fan J.B., Murray S., April C., Belinson H. (2012). Age-dependent brain gene expression and copy number anomalies in autism suggest distinct pathological processes at young versus mature ages. PLoS Genet..

[61] Ka M., Kim W.Y. (2018). ANKRD11 associated with intellectual disability and autism regulates dendrite differentiation via the BDNF/TrkB signaling pathway. Neurobiol. Dis..

[62] Hiraide T., Hattori A., Ieda D., Hori I., Saitoh S., Nakashima M., Saitsu H. (2019). De novo variants in SETD1B cause intellectual disability, autism spectrum disorder, and epilepsy with myoclonic absences. Epilepsia Open.

[63] Fang C.Y., Lai T.C., Hsiao M., Chang Y.C. (2020). The diverse roles of tao kinases in health and diseases. Int. J. Mol. Sci..

[64] Yadav S., Oses-Prieto J.A., Peters C.J., Zhou J., Pleasure S.J., Burlingame A.L., Jan L.Y., Jan Y.N. (2017). TAOK2 Kinase Mediates PSD95 Stability and Dendritic Spine Maturation through Septin7 Phosphorylation. Neuron.

[65] Kahen E.J., Brohl A., Yu D., Welch D., Cubitt C.L., Lee J.K., Chen Y., Yoder S.J., Teer J.K., Zhang Y.O. (2018). Neurofibromin level directs RAS pathway signaling and mediates sensitivity to targeted agents in malignant peripheral nerve sheath tumors. Oncotarget.

[66] Haebich K.M., Pride N.A., Walsh K.S., Chisholm A., Rouel M., Maier A., Anderson V., Barton B., Silk T., Korgaonkar M. (2019). Understanding autism spectrum disorder and social functioning in children with neurofibromatosis type 1: Protocol for a cross-sectional multimodal study. BMJ Open.

[67] Weber J.D., Gutmann D.H. (2012). Deconvoluting mTOR biology. Cell Cycle.

[68] Ganesan H., Balasubramanian V., Iyer M., Venugopal A., Subramaniam M.D., Cho S.G., Vellingiri B. (2019). mTOR signalling pathway—A root cause for idiopathic autism?. BMB Rep..

[69] Rosina E., Battan B., Siracusano M., Di Criscio L., Hollis F., Pacini L., Curatolo P., Bagni C. (2019). Disruption of mTOR and MAPK pathways correlates with severity in idiopathic autism. Transl. Psychiatry.

[70] Xu Z.X., Kim G.H., Tan J.W., Riso A.E., Sun Y., Xu E.Y., Liao G.Y., Xu H., Lee S.H., Do N.Y. (2020). Elevated protein synthesis in microglia causes autism-like synaptic and behavioral aberrations. Nat. Commun..

[71] Monteiro P., Feng G. (2017). SHANK proteins: Roles at the synapse and in autism spectrum disorder. Nat. Rev. Neurosci..

[72] Sato D., Lionel A.C., Leblond C.S., Prasad A., Pinto D., Walker S., O’Connor I., Russell C., Drmic I.E., Hamdan F.F. (2012). SHANK1 deletions in males with autism spectrum disorder. Am. J. Hum. Genet..

[73] Hutsler J.J., Zhang H. (2010). Increased dendritic spine densities on cortical projection neurons in autism spectrum disorders. Brain Res..

[74] Penzes P., Cahill M.E., Jones K.A., Vanleeuwen J.E., Woolfrey K.M. (2011). Dendritic spine pathology in neuropsychiatric disorders. Nat. Neurosci..

[75] Gali Ramamoorthy T., Allen T.J., Davies A., Harno E., Sefton C., Murgatroyd C., White A. (2018). Maternal overnutrition programs epigenetic changes in the regulatory regions of hypothalamic Pomc in the offspring of rats. Int. J. Obes..

[76] Wong C.C.Y., Smith R.G., Hannon E., Ramaswami G., Parikshak N.N., Assary E., Troakes C., Poschmann J., Schalkwyk L.C., Sun W. (2019). Genome-wide DNA methylation profiling identifies convergent molecular signatures associated with idiopathic and syndromic autism in post-mortem human brain tissue. Hum. Mol. Genet..

[77] Chaturvedi P., Tyagi S.C. (2014). Epigenetic mechanisms underlying cardiac degeneration and regeneration. Int. J. Cardiol..

